# Assessing the diagnostic performance of MediXpar texture analysis for C-shaped canal morphology on panoramic radiographs: a pilot study

**DOI:** 10.1007/s00784-026-06830-x

**Published:** 2026-04-06

**Authors:** Sultan Uzun, Samed Satir, Turgut Felek, Guldane Magat

**Affiliations:** 1 Present Address: Department of Oral and Maxillofacial Radiology, Faculty of Dentistry, Bilecik Seyh Edebali University, Pelitozu Mah. Fatih Sultan Mehmet Bulvarı No:27, Merkez/BİLECİK, 11100 Türkiye; 2 Department of Oral and Maxillofacial Radiology, Faculty of Dentistry, Karamanoglu Mehmetbey University, Karaman, Türkiye; 3 Department of Information Technologies, Faculty of Dentistry, Akdeniz University, Antalya, Türkiye; 4 Department of Oral and Maxillofacial Radiology, Faculty of Dentistry, Necmettin Erbakan University, Konya, Türkiye

**Keywords:** C-shaped canal, MediXpar analysis, Digital magnification, Image interpolation, Quantitative texture analysis

## Abstract

**Objectives:**

This study evaluated whether panoramic radiographs could help identify C-shaped canal configurations in mandibular first premolars and offer preliminary diagnostic indicators using the MediXpar bioinformatic platform by examining pixel density and grayscale depth, in a way that approaches the diagnostic value of cone-beam computed tomography (CBCT) while minimizing radiation exposure.

**Methods:**

Panoramic radiographs were retrospectively analyzed with the MediXpar system, a pixel-based quantitative image analysis platform that extracts grayscale density and texture-related parameters from digital radiographs. Three quantitative measures—Xpar, Power Xpar, and Pixel Count—were recorded under zoomed and non-zoomed conditions at two-pixel resolutions (1023 × 496 and 1023 × 593). Logistic regression and receiver operating characteristic (ROC) analyses were used to assess the diagnostic performance of each parameter in identifying C-shaped canals C-shaped canals, with statistical significance set at *p* < 0.05.

**Results:**

Teeth with C-shaped canals showed a significantly larger dentin–pulp complex volume (*p* = 0.001), whereas pulp length did not differ (*p* = 0.653). Digital zoom produced higher pixel-based values (*p* ≤ 0.01), reflecting resampling effects rather than added anatomy. Among all metrics, Power Xpar demonstrated the strongest diagnostic performance (AUC = 0.917 in zoomed and 0.950 in non-zoomed images), showing consistent discrimination across resolutions.

**Conclusions:**

Digital magnification alters the appearance of panoramic images but does not improve diagnostic accuracy. Power Xpar performed best at higher native resolution and remained highly discriminative despite on-screen zoom, indicating that its usefulness depends on true image resolution rather than digital enlargement.

**Clinical relevance:**

Although zooming enhances visual comfort, it may distort quantitative interpretation. MediXpar-based analysis, especially using Power Xpar—offers a practical and reproducible option when CBCT is unavailable.

## Introduction

The root canal system exhibits considerable anatomical variability that may influence success of root canal treatment. Among these variations, the C-shaped canal configuration is clinically significant due to its ribbon-like morphology and complex internal connections resulting from incomplete fusion of Hertwig’s epithelial root sheath [1, 2]. Even though C-shaped canals are most frequently found in mandibular second molars, cone-beam computed tomography (CBCT) studies have confirmed its presence in mandibular first premolars with marked inter-population variability [3–8]. The presence of isthmuses, lamellae, and thin dentinal walls increases procedural difficulty and the risk of perforation, particularly in the so-called “danger zone” [6].

A precise understanding of root canal morphology is essential for achieving successful endodontic outcomes. Over time, various methods (including histologic techniques and radiographic imaging) have been used to investigate root canal configurations [1, 3, 8–11]. With the evolution of imaging technologies, three-dimensional (3D) modalities such as micro-computed tomography (micro-CT) and CBCT have profoundly improved visualization accuracy. Micro-CT is widely regarded as the gold standard for ex vivo investigations due to its micrometer-level resolution and ability to reconstruct high-fidelity 2D and 3D images that capture intricate canal geometry without specimen destruction. These advanced modalities have enabled precise quantitative assessment of canal morphology, dentin thickness, and anatomical variability beyond the capabilities of conventional 2D radiography [12–15].

Although advanced 3D imaging methods have transformed the way root canal systems are studied, 2D techniques such as panoramic radiography are still widely used in everyday dental practice because they offer lower radiation exposure, easy accessibility, and greater cost-efficiency [16]. Digital processing tools, including magnification and contrast enhancement, may improve diagnostic utility [17, 18].

In this context, the MediXpar provides a quantitative image-processing framework that converts pixel density and grayscale depth into numerical parameters, enabling objective and observer-independent radiographic analysis [19]. By translating qualitative radiographic findings into measurable parameters, MediXpar offers a data-driven interpretation of anatomical complexity based on computational morphology. Moreover, the perceptual dimension of digital magnification, unlike optical zoom, which reveals spatial detail, digital magnification reconstructs resolution through pixel interpolation, thus influencing the clinician’s perception of anatomy [20, 21].

Recent studies have explored the utilization of deep learning super-resolution (DL-SR) techniques as options to conventional zoom methods. Demonstrating these advanced algorithms can improve the visual quality of panoramic radiographs more effectively than basic pixel resizing [22]. Deep learning systems that use CBCT have also shown that using 3D data can make 2D panoramic images more useful for diagnosis. This supports the idea that computer techniques can improve low-resolution images [23].

This study explores whether panoramic radiographs can generate measurable texture-based indicators associated with C-shaped canal morphology, thereby supporting diagnostic evaluation and treatment planning. It also examines how MediXpar-derived parameters behave under digital magnification and across different native image resolutions, with particular attention to the stability of quantitative measurements. By analyzing these variables, the study seeks to determine the extent to which texture analysis may reflect internal anatomical complexity beyond subjective visual interpretation.

The MediXpar application, previously evaluated primarily on periapical radiographs [19], is grounded in the principle of X-ray transmissivity. Its application to panoramic radiography represents a methodological extension, enabling assessment of whether texture-derived measurements retain interpretability and practical relevance within this 2D imaging modality.

## Materials and methods

### Study design and ethical approval

This retrospective, observational study was conducted using archived CBCT and panoramic radiographic images obtained between September 2023 and September 2025 from the Department of Oral and Maxillofacial Radiology, Faculty of Dentistry, Necmettin Erbakan University (Konya, Türkiye). The study protocol was approved by the Ethics Committee of Necmettin Erbakan University (Approval No: 2025/669) and was performed in accordance with the ethical standards of the 2013 revision of the Declaration of Helsinki.

An a priori power analysis was performed using G*Power 3.1 (Heinrich Heine University, Düsseldorf, Germany) based on pilot data obtained from an initial subset of panoramic and CBCT images (*n* = 10). The pilot comparison of dentin–pulp complex volume between mandibular first premolars with and without C-shaped canal morphology yielded a large effect size (Cohen’s d = 0.90). Assuming a two-tailed significance level of α = 0.05 and a desired power of 1 − β = 0.80, the minimum total sample size required was calculated as 42 teeth (21 per group).

All CBCT images were acquired using the NewTom Giano (NewTom, Verona, Italy) imaging system. Standardized exposure parameters were applied for all scans: 90 kVp, 8 mA, 12 × 8 cm field of view, 0.2 mm voxel size, and 18 s exposure time. Panoramic radiographic images (PRI) with a resolution of 1023 × 496 pixels were obtained using the Planmeca system (Planmeca, Helsinki, Finland), while those with a resolution of 1023 × 593 pixels were acquired using the NewTom Giano (NewTom, Verona, Italy) system, both with standardized exposure settings of 70 kVp, 8 mA, and 13 s exposure duration.

All images were viewed on a Dell Precision 5820 medical-grade workstation equipped with a 32-inch, 4 K DICOM-calibrated monitor (3840 × 2160 px, 10-bit grayscale depth) under dim ambient light. Monitor luminance and contrast were calibrated monthly to comply with AAPM TG-270 recommendations for diagnostic image viewing [24].

All datasets were exported in Digital Imaging and Communications in Medicine (DICOM) format for standardized analysis and data exchange.

### Sample selection and observer calibration

A total of 301 CBCT scans, representing 602 mandibular first premolars, were retrospectively retrieved from the institutional imaging archive after applying inclusion and exclusion criteria. Eligible cases included patients aged 18 years or older who possessed both right and left mandibular first premolars with no signs of caries, restorations, or previous endodontic treatment. Radiographs displaying motion artifacts, periapical pathology, or developmental anomalies, as well as those from patients undergoing orthodontic treatment or lacking mandibular first premolars, were excluded from the study.

The final dataset was independently assessed by three oral and maxillofacial radiologists with varying levels of clinical experience: Observer 1 (SU, 8 years of experience), Observer 2 (GM, 15 years of experience), and Observer 3 (SS, 16 years of experience). Before the main evaluation, a calibration session was conducted on 20 randomly selected CBCT images to standardize the criteria for identifying canal morphology and assessing image quality. Following calibration, each observer analyzed the full dataset independently, blinded to patient information and to one another’s assessments.

To evaluate reproducibility, each observer re-assessed the same dataset after a 15-day interval under identical viewing conditions. Intraobserver reliability was calculated by comparing each observer’s two assessments, while interobserver reliability was determined by comparing the results among the three observers. The analyses demonstrated excellent intraobserver agreement (ICC = 0.91–0.95) and good interobserver consistency (ICC = 0.84) across all imaging modalities. Cronbach’s α values ranged between 0.87 and 0.93, indicating high internal consistency, while Bland–Altman plots demonstrated good agreement and no evidence of systematic bias between repeated assessments.

### CBCT evaluation and identification of C-shaped canals

CBCT datasets were examined in axial, coronal, and sagittal planes using the NewTom Giano (NewTom, Verona, Italy) imaging system under standardized viewing conditions. Among the 602 mandibular first premolars evaluated; 36 teeth exhibited morphologic features consistent with a C-shaped canal configuration. Identification was based on the presence of a continuous or semicolon-shaped longitudinal groove along the buccal or lingual root surface, visible in at least one axial section.

Each root was assessed at three standardized levels to ensure morphologic accuracy [6]:

- **Coronal third**: 2 mm below the cementoenamel junction (CEJ).
- **Middle third**: midway between the CEJ and the apex.
- **Apical third**: 2 mm above the radiographic apex.

The presence or absence of a C-shaped canal was determined according to the general principles of Fan et al. [11], without further subtyping. This binary classification (“C-shaped canal present” or “absent”) was used to ensure consistent and reproducible categorization across all observers.

**Fig. 1:**
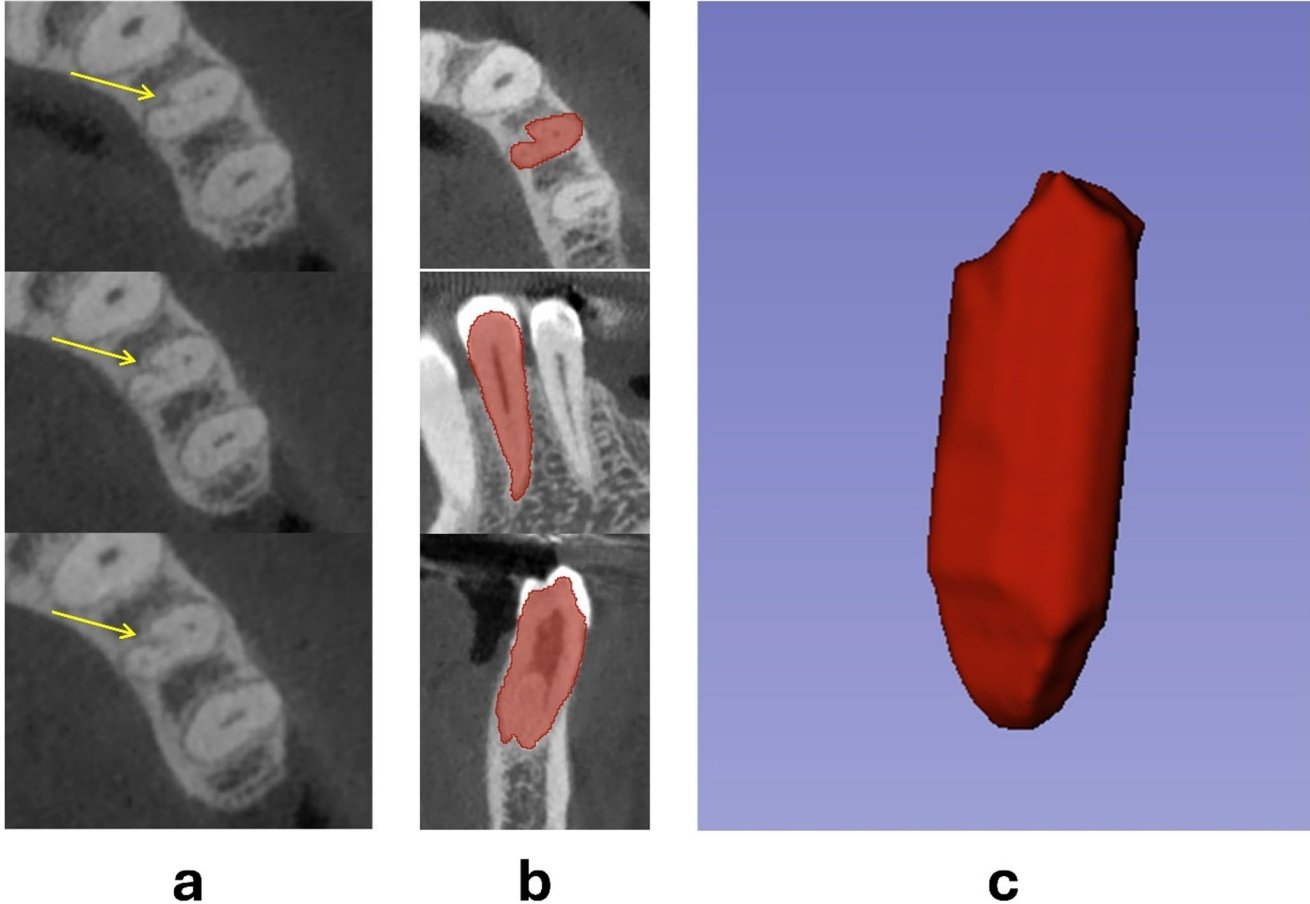
Representative CBCT slices illustrating C-shaped canal morphologies and corresponding 3D reconstruction. (**a**) CBCT axial slices of a C-shaped canal (yellow arrows: radicular groove); (**b**) semi-manual automatic segmentation with thresholding; (**C**) 3D volumetric reconstruction of the dentin–pulp complex

Representative cross-sectional CBCT images demonstrating characteristic C-shaped morphologies were selected for visual validation and presented in Fig. 1. In cases of interpretive discrepancy, consensus on representative images was achieved through joint review sessions.

**Fig. 2:**
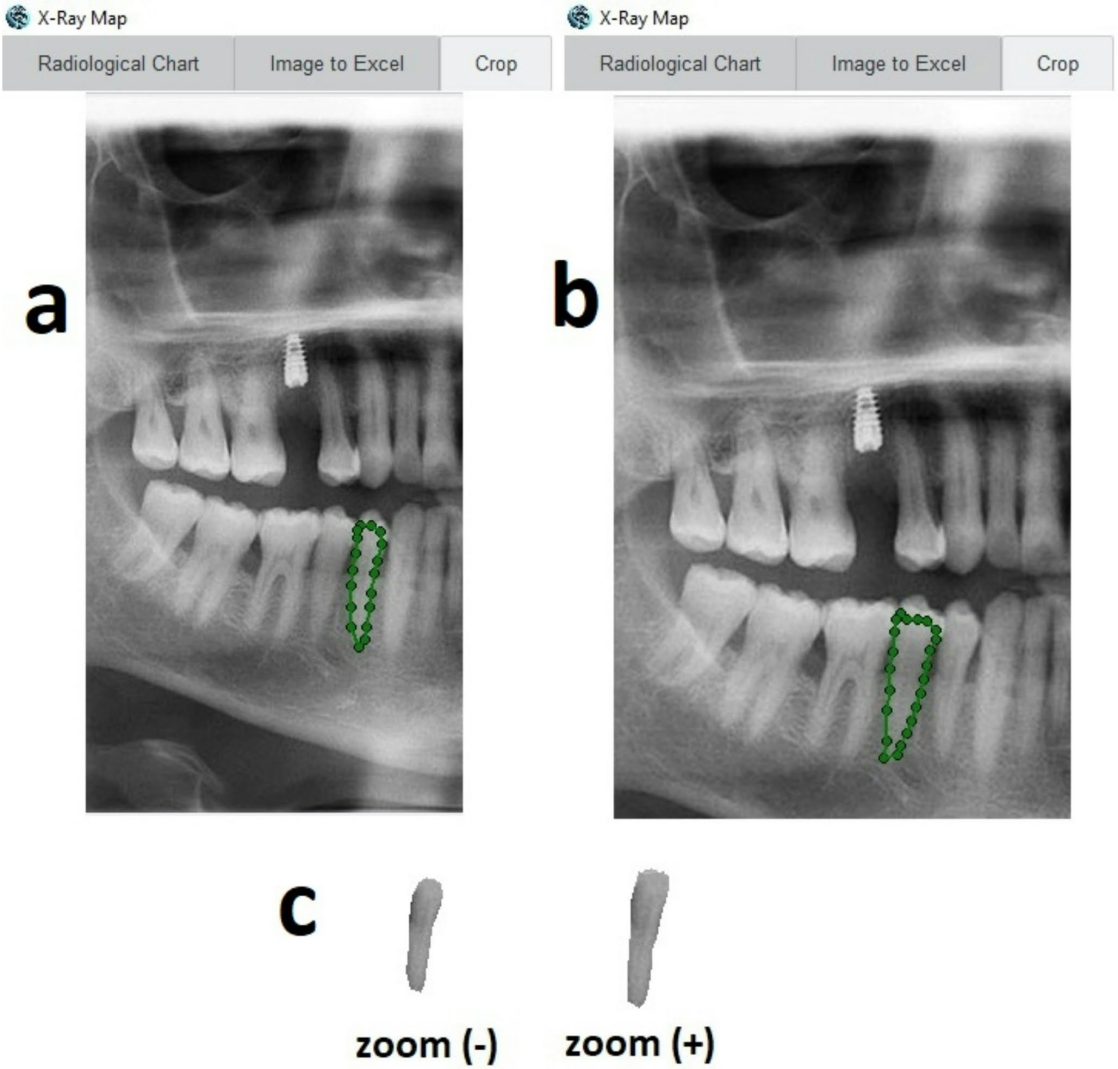
Workflow of the MediXpar analysis. (**a**) ROI selection for a mandibular first premolar without zoom, (**b**) ROI selection for a mandibular first premolar with zoom, (**c**) Final version of the cropped dentin-pulp complex images without and with zoom

### Panoramic radiographic analysis using MediXpar

After CBCT-based identification of C-shaped canal morphology, the institutional archive was reassessed for corresponding 2D radiographs (both panoramic and periapical images). However, the number of diagnostically acceptable periapical radiographs specifically acquired for mandibular first premolars was too limited. Therefore, a comparative three-modality analysis was not feasible. Consequently, the present study focused on panoramic radiographs as the standardized 2D modality available for all included cases.

All panoramic radiographs corresponding to the 72 CBCT-identified mandibular first premolars were retrospectively collected. Following a quality control process, 13 C-shaped and 16 non–C-shaped radiographs exhibiting motion artifacts, inconsistent pixel resolution, or inadequate diagnostic quality were excluded, leaving 43 diagnostically acceptable images for further analysis.

Subsequently, all radiographs were categorized according to their native pixel resolution (hereafter abbreviated as *px*). Two resolution groups were identified, and to ensure demographic and anatomical comparability, cases were matched for age, sex, and tooth position (right or left).

- ***1023 × 496 px***: after these criteria, 10 C-shaped and 16 non–C-shaped radiographs were retained.
- ***1023 × 593 px***: 12 C-shaped and 5 non–C-shaped radiographs were selected.

After the final matching process, the dataset comprised 43 panoramic radiographs—22 with C-shaped canals and 21 without—which were subsequently analyzed using the MediXpar software. Accordingly, the present study met the sample size requirement determined by power analysis.

Quantitative texture parameters (Xpar, Power Xpar, Pixel Count) were extracted from panoramic radiographs using MediXpar software (https://mediterraneanradiology.com/) [19]. Unlike conventional histogram-based texture metrics, MediXpar employs a physics-informed model that integrates grayscale entropy with transmittance variance, allowing the quantification of spatial gray-level energy as a function of anatomical density gradients. This approach enables more physiologically meaningful interpretation of pixel variation across dental hard tissues.

$$Rij=[(log((Ai-Bij)^2+1)\times sin(\pi/2\times Bij/Ai))^2]$$

Following this formulation, the key components of the equation can be clarified to support interpretability:

- Rij – the resulting calculated value for the pixel at row *i* and column *j*, representing depth (z-value).
- Ai – the maximum pixel value in the *i*-th row, used as a reference point for normalization.
- Bij – the pixel value at the *i*-th row and *j*-th column, representing grayscale intensity at that specific location [19].

This algorithm models x-ray transmittance through mineralized tissues, reconstructing a depth map that reflects internal structural heterogeneity. Images were cropped to the region of interest which included only dentin and pulp using the Pillow library. Edge detection was achieved via the Sobel operator implemented in *scipy.ndimage*, and matrices were processed with NumPy and visualized using Plotly in 2D.

For each panoramic radiograph, quantitative evaluation was performed using the MediXpar software. The following parameters [19] were automatically calculated and recorded for both zoomed and unzoomed conditions:

1. **Total pixel count**: the total number of pixels within the selected region of interest (ROI) on the panoramic radiograph, representing the spatial resolution and sampling density of the analyzed area. The zoomed condition was applied digital magnification through pixel interpolation, whereas the unzoomed condition retained the image’s native resolution.
2. **Xpar depth index**: a transmittance-based computational variable expressing the relative depth variation per pixel, derived from the equation.

$${R}_{ij}={[\mathrm{l}\mathrm{o}\mathrm{g}(({A}_{i}-{B}_{ij}{)}^{2}+1)\times\mathrm{s}\mathrm{i}\mathrm{n}\left(\frac{\pi}{2}\frac{{B}_{ij}}{{A}_{i}}\right)]}^{2}$$

where ${A}_{i}$denotes the maximum grayscale value in each row and ${B}_{ij}$the grayscale value at pixel $(i,j)$. This index estimates 3D morphological heterogeneity; irregular values suggest greater internal structural complexity such as bifurcation or irregular canal outlines.

3. **Power Xpar depth index**: A parameter generated by exponential computation from the distribution and magnitude of Xpar depth values ​​across the ROI, which presents pixel variation more clearly. Increasing power metric values ​​can identify anatomical complexities such as C-shaped canal configurations.

Together, these parameters can describe the pixel-based morphological variations within radiography, enabling the indirect quantitative assessment of internal structure from 2D images.

### Volumetric validation

Volumetric validation was performed to establish the relationship between pixel-based radiographic parameters derived from MediXpar analysis and internal morphologic characteristics of the teeth. A total of 43 mandibular first premolars (22 with C-shaped canals and 21 without) were included in this stage. All CBCT datasets were exported in DICOM format and analyzed using 3D Slicer software (version 5.6.2; www.slicer.org).

The entire dentin-pulp complex was manually segmented as the region of interest (ROI) across the axial, coronal, and sagittal planes to create volume with the Segment Editor module. Semi-automatic segmentation was then performed using the Threshold and Paint tools, followed by manual improvement to ensure detailed anatomical delineation. Gray-level density differences between enamel and underlying dentin were used to exclude enamel and include the dentin-pulp-cementum complex [25].

When automatic segmentation presented ambiguous borders (particularly at the enamel–dentin junction) manual corrections were applied slice-by-slice in all spatial planes to achieve reliable definition. Post-processing steps were performed within the *Segment Editor* to enhance segmentation quality: a Smoothing effect (median filter, kernel size = 0.5 mm) minimized surface irregularities, the Islands → Remove small islands function eliminated voxel-level noise, and a morphological closing operation (Wrap Solidify) was applied to fill minor discontinuities and maintain 3D surface continuity.

Final volumes were computed using the *Segment Statistics* module and expressed in cubic millimeters (mm³). Representative 3D reconstructions illustrating both C-shaped and non–C-shaped canal morphologies are presented in Fig. 1.

To ensure the reliability of the volumetric data, all segmentations were performed independently by two experienced observers (SU, SS). To assess intraobserver reliability, both observers repeated the entire segmentation procedure (including both manual and semi-automatic steps) for the complete dataset after a one-week interval, under identical viewing conditions. Interobserver and intraobserver agreement for the volumetric measurements were evaluated using the intraclass correlation coefficient (ICC). The analysis demonstrated excellent reliability, with intraobserver ICC values of 0.87 for both observers and an interobserver ICC of 0.92, indicating high consistency of the manual–semi-automatic segmentation protocol.

### Statistical analysis

Data was analyzed using IBM SPSS Statistics (version 27; IBM Corp., Armonk, NY, USA). Normality of continuous variables was assessed using the Shapiro–Wilk test. Depending on data distribution, independent-samples t-test or Mann–Whitney U test was applied to compare morphometric and volumetric parameters between C-shaped and non–C-shaped canals. Differences between zoomed and non-zoomed analyses were evaluated using the Wilcoxon signed-rank test for paired samples. Comparisons among multiple pixel-dependent parameters (e.g., Xpar, Power Xpar, Pixel Count) were conducted using Kruskal–Wallis or one-way ANOVA tests, followed by post-hoc pairwise comparisons with Bonferroni correction where applicable. Correlations between quantitative variables were analyzed using Spearman’s rank correlation coefficient. Diagnostic performance of Power Xpar and related MediXpar metrics for detecting C-shaped canal morphology was assessed by receiver operating characteristic (ROC) analysis, with area under the curve (AUC) values reported. All tests were two-tailed, and a p-value < 0.05 was considered statistically significant.

## Results

A total of 43 CBCT scans were analyzed, comprising 22 teeth with and 21 without a C-shaped canal morphology. The study population included 14 females (32.6%) and 29 males (67.4%) aged 17–54 years. Twenty-four participants were between 17 and 39 years of age, and 19 were between 40 and 54 years. Panoramic radiography images were acquired using two resolutions: 1023 × 496 pixels (*n* = 26) and 1023 × 593 pixels (*n* = 17). These descriptive characteristics are presented in Table 1.

**Table 1:** Descriptive statistics of dentin-pulp complex and pulp length according to gender, age groups, C-canal presence, and pixel size

**Descriptive statistics of dentin-pulp complex**							
**Variable**	**Group**	***n***	**Minimum- Maximum**	**Mean (Median) ± SD**	**95% CI**	**Effect size**	***p*** **-value**
Gender	Female	14	334.67–565.10	399.79 (384.63) ± 54.91	[368.09–431.49]	*r* = 0.345	0.072
	Male	29	240.76–613.75	428.23 (425.25) ± 81.47	[397.24–459.22]		
Age groups	18–39 years	24	240.76–464.75	391.10 (384.63) ± 55.82	[367.53–414.67]	*r* = 0.421	0.020 *
	40–54 years	19	362.22–613.75	454.18 (412.91) ± 81.25	[415.02–493.34]		
C-canal presence	None	21	240.76–545.50	383.86 (393.64) ± 58.03	[357.44–410.27]	*r* = 0.593	0.001 **
	Yes	22	340.74–613.75	452.48 (446.36) ± 73.91	[419.71–485.26]		
Pixel size	1023 × 496	26	240.76–613.75	404.92 (394.61) ± 72.37	[375.68–434.15]	*r* = 0.299	0.104
	1023 × 593	17	334.67–610.02	440.46 (425.25) ± 74.47	[402.17–478.75]		
**Descriptive statistics of pulp length**							
**Variable**	**Group**	**n**	**Minimum–Maximum**	**Mean (Median) ± SD**	**95% CI**	**Effect size**	**p-value**
Gender	Female	14	19.65–23.35	21.73 (21.64) ± 1.14	[21.07–22.39]	*r* = − 0.089	0.650
	Male	29	19.21–24.90	21.69 (21.43) ± 1.53	[21.11–22.27]		
Age groups	18–39 years	24	19.21–22.60	21.33 (21.35) ± 0.82	[20.99–21.68]	*r* = 0.307	0.089
	40–54 years	19	20.60–24.90	22.17 (22.10) ± 1.82	[21.29–23.04]		
C-canal presence	None	21	19.21–23.86	21.73 (21.52) ± 1.22	[21.17–22.29]	*r* = − 0.082	0.653
	Yes	22	19.60–24.90	21.67 (21.35) ± 1.58	[20.97–22.38]		
Pixel size	1023 × 496	26	19.21–24.90	21.76 (21.50) ± 1.43	[21.19–22.34]	*r* = − 0.045	0.813
	1023 × 593	17	19.70–23.50	21.61 (21.40) ± 1.40	[20.89–22.33]		

*n* Number, *SD* Standard deviation, %: Percent, *Cl* Confidence interval, *r* rank biserial correlation coefficient (effect size). *: *p* < 0.05. **: *p* < 0.01

Quantitative image-derived parameters included dentin-pulp complex volume, pulp length, Xpar, Power Xpar, and Pixel Count, each assessed under zoomed and non-zoomed conditions when applicable. Descriptive and comparative results are summarized in Tables 1, 2 and 3. No statistically significant differences were observed between sexes or age groups for any of the evaluated variables (all *p* > 0.05; see Tables 1 and 2 for exact values). Similarly, comparisons according to C-canal presence revealed a significant difference in pulp volume (*p* = 0.001, *r* = 0.593), with higher values observed in C-shaped premolars compared to non-C-shaped ones. No statistically significant differences were found for other morphometric parameters. Under the zoomed condition, both Xpar and Power Xpar values increased systematically with higher image resolution (1023 × 593 pixels), suggesting that pixels enhance the detection of gray-level variability within the region of interest. Although mean and median trends were directionally consistent between groups, substantial interindividual variability was observed, as reflected by broad interquartile ranges (Tables 1 and 2).

**Table 2:** Descriptive statistics of MediXpar analysis

**Descriptive statistics of Xpar (Zoomed)**							
**Variable**	**Group**	***n***	**Minimum–Maximum**	**Mean (Median) ± SD**	**95% CI**	**Effect size**	***p*** **-value**
Gender	Female	14	8.30–19.52	12.67 (12.14) ± 3.19	[10.83–14.51]	*r* = 0.081	0.678
	Male	29	7.15–21.43	13.35 (12.22) ± 3.67	[11.95–14.74]		
Age groups	18–39 years	24	7.15–21.43	13.66 (12.22) ± 4.27	[11.85–15.46]	*r* = − 0.096	0.599
	40–54 years	19	8.70–18.34	12.45 (12.13) ± 2.09	[11.45–13.46]		
C-canal presence	None	21	7.15–21.43	13.27 (12.13) ± 3.94	[11.47–15.06]	*r* = 0.015	0.942
	Yes	22	7.50–18.72	12.99 (12.23) ± 3.10	[11.61–14.37]		
Pixel size	1023 × 496	26	7.15–18.34	12.53 (11.63) ± 3.67	[11.05–14.01]	*r* = 0.373	**0.042** *
	1023 × 593	17	8.34–21.43	14.04 (13.66) ± 3.11	[12.44–15.63]		
**Descriptive statistics of Xpar (Non-Zoomed)**							
**Variable**	**Group**	**n**	**Minimum–Maximum**	**Mean (Median) ± SD**	**95% CI**	**Effect size**	**p-value**
Gender	Female	14	8.00–20.33	13.18 (12.71) ± 3.28	[11.28–15.07]	*r* = 0.266	0.166
	Male	29	8.85–21.36	14.42 (13.90) ± 3.31	[13.16–15.67]		
Age groups	18–39 years	24	8.85–21.36	14.47 (13.18) ± 3.89	[12.83–16.11]	*r* = − 0.077	0.678
	40–54 years	19	9.50–18.89	13.44 (12.94) ± 2.36	[12.30–14.58]		
C-canal presence	None	21	8.85–21.36	14.11 (13.65) ± 3.22	[12.65–15.58]	*r* = − 0.093	0.610
	Yes	22	9.50–19.36	13.92 (12.94) ± 3.47	[12.38–15.46]		
Pixel size	1023 × 496	26	8.85–19.36	13.50 (12.87) ± 3.50	[12.09–14.91]	*r* = 0.314	0.087
	1023 × 593	17	9.80–21.36	14.80 (14.49) ± 2.93	[13.29–16.30]		
**Descriptive statistics of Power Xpar (Zoomed)**							
**Variable**	**Group**	**n**	**Minimum–Maximum**	**Mean (Median) ± SD**	**95% CI**	**Effect size**	**p-value**
Gender	Female	14	103.57–152.83	127.73 (122.89) ± 13.90	[119.71–135.76]	*r* = 0.256	0.182
	Male	29	104.22–165.34	134.28 (136.89) ± 17.27	[127.71–140.85]		
Age groups	18–39 years	24	104.22–165.34	133.38 (131.28) ± 17.60	[125.95–140.81]	*r* = − 0.110	0.549
	40–54 years	19	111.45–154.17	130.58 (127.32) ± 15.03	[123.34–137.83]		
C-canal presence	None	21	104.22–165.34	132.45 (128.29) ± 17.82	[124.34–140.56]	*r* = 0.017	0.932
	Yes	22	111.45–154.17	131.86 (125.12) ± 15.29	[125.08–138.64]		
Pixel size	1023 × 496	26	104.22–165.34	138.28 (138.31) ± 17.91	[131.05–145.52]	*r* = − 0.520	**0.004** **
	1023 × 593	17	111.45–136.89	122.76 (122.79) ± 6.92	[119.20–126.32]		
**Descriptive statistics of Power Xpar (Non-Zoomed)**							
**Variable**	**Group**	**n**	**Minimum–Maximum**	**Mean (Median) ± SD**	**95% CI**	**Effect size**	**p-value**
Gender	Female	14	91.12–152.83	126.80 (122.82) ± 13.37	[119.08–134.52]	*r* = 0.281	0.143
	Male	29	104.22–174.97	133.67 (134.20) ± 16.35	[127.45–139.89]		
Age groups	18–39 years	24	104.22–174.97	133.20 (133.06) ± 16.35	[126.29–140.10]	*r* = − 0.162	0.372
	40–54 years	19	91.12–154.17	129.21 (126.00) ± 14.80	[122.07–136.34]		
C-canal presence	None	21	91.12–174.97	131.28 (126.07) ± 17.67	[123.24–139.32]	*r* = 0.065	0.725
	Yes	22	104.22–154.17	131.58 (127.58) ± 13.82	[125.45–137.71]		
Pixel size	1023 × 496	26	104.22–174.97	137.75 (136.51) ± 16.63	[131.03–144.46]	*r* = − 0.606	**0.001** **
	1023 × 593	17	91.12–136.51	121.78 (121.99) ± 6.73	[118.32–125.24]		
**Descriptive statistics of Pixel Count (Non-Zoomed)**							
**Variable**	**Group**	**n**	**Minimum–Maximum**	**Mean (Median) ± SD**	**95% CI**	**Effect size**	**p-value**
Gender	Female	14	1200–2600	1905.21 (1848.00) ± 435.72	[1653.64–2156.79]	*r* = 0.140	0.468
	Male	29	1400–2700	2017.86 (2030.00) ± 428.02	[1855.05–2180.67]		
Age groups	18–39 years	24	1300–2600	1950.42 (1881.50) ± 425.41	[1770.78–2130.05]	*r* = 0.154	0.399
	40–54 years	19	1400–2700	2020.05 (2133.00) ± 441.20	[1807.40–2232.70]		
C-canal presence	None	21	1300–2700	1992.95 (1976.00) ± 415.25	[1803.93–2181.97]	*r* = − 0.078	0.671
	Yes	22	1200–2500	1969.95 (1896.00) ± 450.57	[1770.18–2169.73]		
Pixel size	1023 × 496	26	1300–2700	2015.58 (2050.00) ± 446.67	[1835.16–2195.99]	*r* = − 0.152	0.412
	1023 × 593	17	1200–2600	1928.59 (1846.00) ± 407.12	[1719.26–2137.91]		
**Descriptive statistics of Pixel Count (Zoomed)**							
**Variable**	**Group**	**n**	**Minimum–Maximum**	**Mean (Median) ± SD**	**95% CI**	**Effect size**	**p-value**
Gender	Female	14	1700–4200	2828.29 (2540.50) ± 797.97	[2367.55–3289.02]	*r* = 0.273	0.154
	Male	29	1800–4600	3150.86 (3010.00) ± 740.21	[2869.30–3432.42]		
Age groups	18–39 years	24	1700–3940	2835.62 (2730.00) ± 603.10	[2580.96–3090.29]	*r* = 0.344	0.056
	40–54 years	19	1900–4600	3311.37 (3570.00) ± 876.73	[2888.80–3733.94]		
C-canal presence	None	21	1700–4200	2972.86 (2822.00) ± 793.57	[2611.63–3334.09]	*r* = 0.128	0.481
	Yes	22	1900–4600	3115.50 (3074.50) ± 749.23	[2783.31–3447.69]		
Pixel size	1023 × 496	26	1700–4600	2998.04 (2787.00) ± 782.24	[2682.09–3313.99]	*r* = 0.109	0.559
	1023 × 593	17	1900–4200	3118.94 (3069.00) ± 756.40	[2730.04–3507.85]		

*n* Number, *SD *Standard deviation, %: Percent, *Cl *Confidence interval, *r* rank biserial correlation coefficient (effect size). *p < 0.05; **p < 0.01

**Table 3:** Comparison of zoomed and non-zoomed measurements according to c canal presence at different pixel sizes

Pixel size	C canal presence	Parameter	Zoomed mean ± SD (Median [IQR])	Non-zoomed mean ± SD (Median [IQR])	Z	*p*-value
**1023 × 496**	No	Xpar	12.90 ± 4.14 (11.78 [10.64–14.15])	13.70 ± 3.54 (12.93 [11.36–14.94])	-2.172	0.029 *
		Power Xpar	137.18 ± 17.92 (138.31 [125.44–148.67])	136.15 ± 17.55 (135.41 [125.03–148.41])	-2.068	0.039 *
		Pixel Count	2876.25 ± 650.83 (2787.00 [2430.00–3532.50])	1965.69 ± 409.53 (1946.50 [1745.25–2249.00])	-3.516	**< 0.001** **
	Yes	Xpar	11.93 ± 2.84 (11.43 [10.46–12.24])	13.19 ± 3.59 (12.59 [11.33–13.71])	-2.293	**0.020** *
		Power Xpar	140.05 ± 18.72 (140.71 [132.58–151.17])	140.31 ± 15.59 (140.42 [131.75–147.04])	-1.784	0.084
		Pixel Count	3192.90 ± 961.87 (3003.00 [2445.00–4070.00])	2095.40 ± 513.00 (2122.00 [1683.00–2508.50])	-2.803	**0.002** *
**1023 × 593**	No	Xpar	14.44 ± 3.33 (12.22 [12.13–16.97])	15.44 ± 1.33 (14.81 [14.49–16.50])	-1.214	0.312
		Power Xpar	117.31 ± 2.62 (117.26 [115.92–118.70])	115.70 ± 2.87 (117.24 [113.84–117.83])	-1.753	0.125
		Pixel Count	3282.00 ± 1184.72 (2938.00 [2225.00–4472.00])	2080.20 ± 469.81 (2146.00 [1748.00–2325.00])	-2.023	0.062
	Yes	Xpar	13.87 ± 3.14 (13.79 [11.96–14.73])	14.53 ± 3.40 (13.64 [12.04–15.73])	-1.490	0.151
		Power Xpar	125.03 ± 6.93 (123.35 [122.14–124.83])	124.31 ± 6.25 (122.90 [121.38–125.45])	-1.883	0.064
		Pixel Count	3051.00 ± 552.00 (3074.50 [2702.25–3316.50])	1865.42 ± 382.13 (1781.00 [1626.50–1933.75])	-3.059	**< 0.001** **

*SD *Standard deviation, *IQR *Interquartile range, *Z *standardized test statistics (Wilcoxon signed-rank test); *p < 0.05; **p < 0.01

Within-subject differences between zoomed and non-zoomed measurements are summarized in Table 3. The Wilcoxon signed-rank test demonstrated significant differences in Pixel Count for both pixel sizes (*p* ≤ 0.01), with zoomed images consistently yielding higher pixel counts. At 1023 × 496 pixels, the Xpar parameter also differed significantly between zoomed and non-zoomed images in cases without a C-canal (Z = − 2.389, *p* = 0.017). For Power Xpar, differences were generally smaller, reaching statistical significance only in selected strata (Z ≈ − 2.519, *p* = 0.012).

**Fig. 3:**
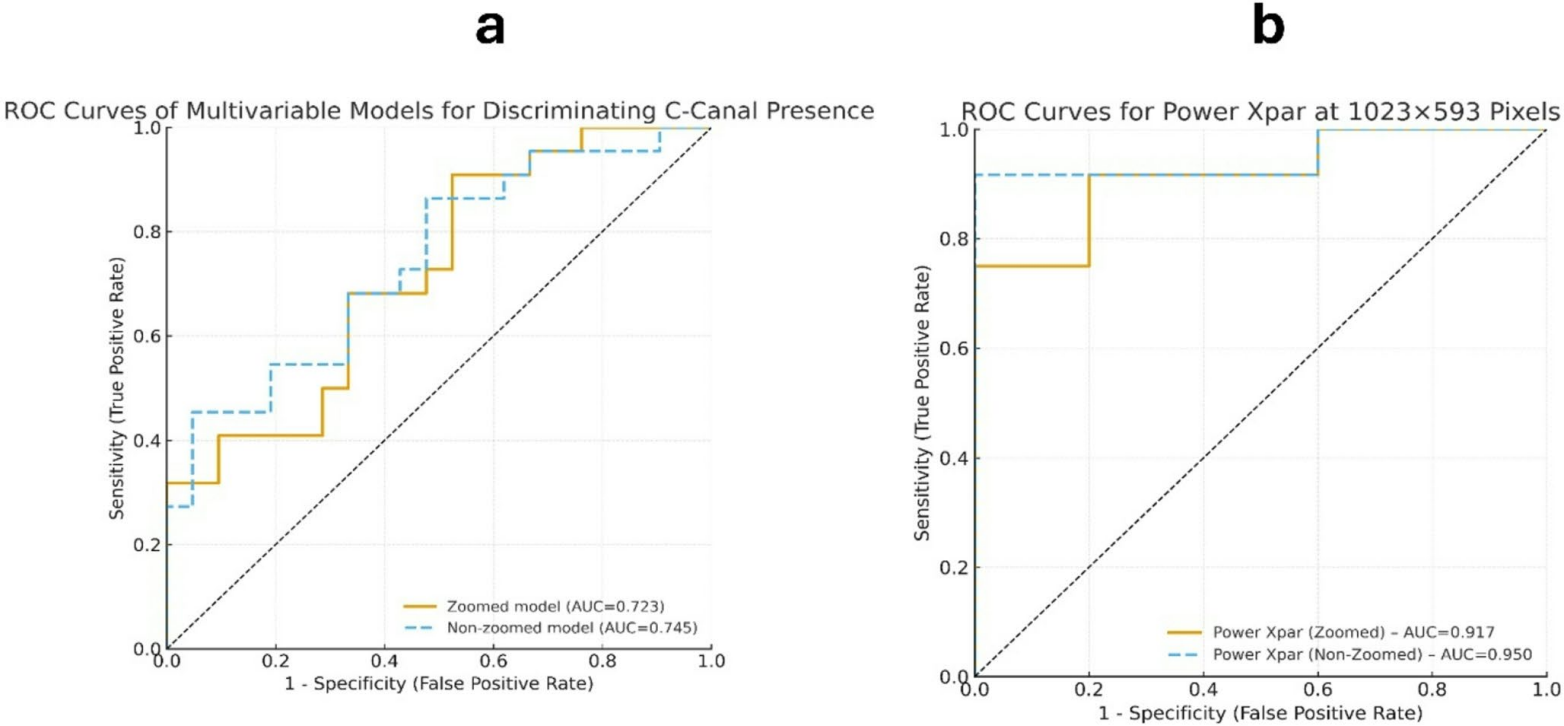
ROC analysis for the prediction of C-shaped canal morphology (**a**) ROC curves of multivariable logistic regression models predicting the presence of a C-shaped canal (**b**) ROC curves illustrating the diagnostic performance of image-derived parameters, stratified by pixel size and imaging condition

Multivariable logistic regression models assessing the diagnostic performance for the presence of a C-shaped canal are summarized in Table 4. Model A1, which included dentin-pulp complex volume, pulp length, Xpar, Power Xpar, and Pixel Count measured under the zoomed condition, achieved an AUC of 0.723, indicating fair discriminatory ability. Among all variables, pixel size was the only variable significantly associated with C-canal presence (*p* < 0.05). Model A2, based on the non-zoomed parameters, demonstrated a comparable discrimination level with an AUC of 0.745, and pixel size again emerged as the only significant independent variable (*p* < 0.05). The combined model (A3), which integrated all zoomed and non-zoomed parameters along with sex and age, exhibited the highest overall discriminative performance (AUC = 0.779, 95% CI 0.640–0.918). However, the overall model fit was not statistically significant (LR χ²(9) = 10.67, *p* = 0.299), indicating limited incremental benefit from combining multiple parameters. The adjusted odds ratios and 95% confidence intervals for each covariate are provided in Table 5, and the ROC curves for the zoomed and non-zoomed models are shown in Fig. 3, demonstrating comparable discrimination (AUC = 0.723 vs. 0.745).

**Table 4:** Multivariable logistic regression associated with C-canal presence (Zoomed vs. Non-zoomed)

Model	Predictor	OR	95% CI	*p*-value
**A1 – zoomed**	Xpar (zoomed)	0.638	0.300–1.356	0.243
	Power Xpar (zoomed)	1.311	0.626–2.743	0.473
	Pixel Count (zoomed)	1.322	0.621–2.813	0.469
	Pixel Size	2.700	1.188–6.136	0.018*
	Gender	1.462	0.717–2.980	0.297
	Age Group	0.692	0.326–1.470	0.297
**A2 – non-zoomed**	Xpar (non-zoomed)	0.660	0.310–1.406	0.282
	Power Xpar (non-zoomed)	1.584	0.721–3.482	0.252
	Pixel Count (non-zoomed)	1.075	0.535–2.160	0.840
	Pixel Size	3.048	1.280–7.256	0.012*
	Gender	1.553	0.745–3.237	0.240
	Age Group	0.785	0.393–1.569	0.494
**A3-combined model (zoomed + non-zoomed)**	Xpar, Power Xpar, Pixel Count; plus Pixel size, Gender, Age group			LR χ²(9) = 10.67, *p* = 0.299†

pixel size (0 = 1023 × 496, 1 = 1023 × 593); gender (1 = female, 2 = male); age group (1 = 17–39, 2 = 40–54). OR=odds ratio; CI=confidence interval. † p-value for overall model fit from likelihood-ratio test vs. intercept-only model. Overall performance: A1 AUC = 0.723; A2 AUC = 0.745; A3 AUC = 0.779 (95% CI 0.640–0.918). Composite score (per 1 SD): OR = 3.24 (1.43–7.38), *p* = 0.005. *:*p* < 0.05

**Table 5:** ROC-based performance of measurements for discriminating C-canal presence, stratified by pixel size (zoomed and non-zoomed)

Pixel size	Metric	AUC	Optimal threshold	Sensitivity	Specificity	Youden J
**1023 × 496**	Xpar (zoomed)	0.438	<= 12.75	0.900	0.310	0.210
	Power Xpar (zoomed)	0.538	>= 131.15	0.800	0.380	0.180
	Pixel Count (zoomed)	0.594	>= 3680.00	0.400	0.940	0.340
	Xpar (non-zoomed)	0.450	<= 12.75	0.600	0.620	0.230
	Power Xpar (non-zoomed)	0.575	>= 129.58	0.900	0.380	0.270
	Pixel Count (non-zoomed)	0.597	>= 2070.00	0.700	0.620	0.320
**1023 × 593**	Xpar (zoomed)	0.467	<= 15.89	0.920	0.400	0.320
	Power Xpar (zoomed)	**0.917**	>= 122.79	0.750	1.000	0.750
	Pixel Count (zoomed)	0.483	<= 3960.00	1.000	0.400	0.400
	Xpar (non-zoomed)	0.333	<= 14.15	0.580	1.000	0.580
	Power Xpar (non-zoomed)	**0.950**	>= 119.07	0.920	1.000	0.920
	Pixel Count (non-zoomed)	0.333	<= 2133.00	0.920	0.600	0.520

*AUC* Area under the curve

ROC analyses stratified by pixel size and imaging condition are presented in Table 5 and illustrated in Fig. 3. Among all evaluated parameters, Power Xpar demonstrated the highest diagnostic performance in distinguishing the presence of a C-shaped canal, particularly under the non-zoomed condition. At a resolution of 1023 × 593 pixels, the zoomed Power Xpar yielded an AUC of 0.917, with an optimal cutoff value of ≥ 122.79 corresponding to a sensitivity of 0.75, specificity of 1.00, and Youden J index of 0.75. The non-zoomed Power Xpar achieved an influence success even higher AUC of 0.950, with a threshold value of ≥ 119.07, sensitivity of 0.92, specificity of 1.00, and Youden J of 0.92. These findings indicate that Power Xpar, particularly in the zoomed condition, provided excellent discriminative capacity for identifying C-shaped canal morphology at higher image resolutions. All other parameters showed poor-to-fair diagnostic performance (AUC range, 0.44–0.60), underscoring the superior discriminatory power of Power Xpar across pixel sizes. Figure 3 graphically depicts these findings, emphasizing the consistently higher ROC curve of Power Xpar compared with the other evaluated parameters.

## Discussion

The C-shaped canal morphology is believed to have a worldwide prevalence of 10% [6], but the prevalence is higher in East Asian populations (31.5% in Chinese and Japanese [26] and 32.7% in the Korean population [27] while rates in Europe, Africa, and North America remain between 3% and 7% [27, 28, 29]. Such pronounced geographic variation suggests that the development of C-shaped canals may be influenced by ethnic, genetic, and developmental factors. In the present dataset, C-shaped canal morphology was identified in 36 of 602 mandibular first premolars, corresponding to an incidence of approximately 6%. This rate aligns with the lower prevalence reported in European and Middle Eastern populations and contrasts sharply with the much higher rates documented in East Asian cohorts. Previous studies from Türkiye have likewise reported similarly low frequencies of this morphology, particularly in mandibular first premolars, where prevalence has consistently remained below 10% [6]. The relative rarity of C-shaped canals in the Turkish population limits the availability of radiographic data and poses challenges for conducting large-sample morphological analyses. Beyond the epidemiological variance, ethnic variability may also influence the internal geometric complexity and mineralization patterns of these canals, which directly affects their radiographic density profiles. Given this rarity, early and precise identification of C-shaped canal configurations becomes crucial for successful endodontic management. Recognizing these variations before treatment can help clinicians anticipate procedural challenges, select appropriate imaging modalities, and achieve more predictable clinical outcomes while minimizing unnecessary radiation exposure. the Power Xpar thresholds identified in this Turkish cohort provide a robust diagnostic baseline, however, the inherent ethnic diversity in root canal systems suggests that these quantitative indicators should be further validated in high-prevalence populations to ensure global diagnostic reliability.

Most of the literature on C-shaped canal morphology encompasses previous investigations based on CBCT or micro-CT imaging and is limited to clinical settings where these advanced imaging modalities are routinely available [2, 6, 11, 25, 30–32]. However, the translation of root canal variations into 2D radiographic diagnostic methods has been largely unexplored. The current study aimed to address this gap by evaluating whether quantitative, pixel-based parameters obtained from panoramic radiographs could reflect the C-shaped canal configurations of mandibular premolars. This research bridges the interpretation gap between 3D imaging and commonly used 2D modalities by evaluating how MediXpar-derived grayscale metrics reflect underlying CBCT-validated volumetric differences. A significant moderate assosiaction was observed between dentin-pulp complex volume and age groups (*r* = 0.421, *p* = 0.020), reflecting age-related biological variability in internal root anatomy. The subsequent analyses therefore examined whether grayscale-derived pixel parameters could serve as indirect indicators of these volumetric differences. Previous panoramic studies investigating C-shaped canals have primarily relied on visual pattern recognition, reporting that conical-shaped root configurations could predict C-shaped morphology with reasonable sensitivity and specificity. In contrast, the present study advances this approach by introducing a quantitative, pixel-based framework aimed at capturing grayscale variability potentially associated with underlying 3D anatomical differences. This shift from qualitative observation to computational interpretation demonstrates that even conventional panoramic radiographs, when analyzed through MediXpar, can provide measurable image-based markers related to C-shaped morphology [8]. The findings thus contribute a novel bioinformatic perspective to endodontic imaging, suggesting that conventional panoramic radiographs, when analyzed computationally, may offer supportive information for identifying canal variation.

The present results revealed that there is significant correlation between volumetric and longitudinal morphometrics of the mandibular first premolar. Dentin-pulp complex volume increased significantly with age (*p* = 0.020), while pulp length remained constant, indicating that mineral apposition and canal elongation are governed by different biological processes. This pattern is consistent with previous CBCT-based studies [33, 34] showing that secondary dentin deposition primarily modifies canal width rather than its axial extent. The greater volumes observed in C-shaped teeth substantiate the morphologic implications of radicular fusion, in agreement with Fernandes et al. [2] and Jin et al. [35], where enlarged buccolingual and mesiodistal dimensions characterize fused-root anatomy. The lack of a significant difference in pulp length, despite notable volumetric enlargement, could indicate that the enlargement occurs mainly in the lateral dimension rather than through apico-coronal elongation.

Clinically, this finding may underscore that volumetric asymmetry, rather than canal length, may be a more reliable radiographic sign for detecting complex root systems. These results reinforce the notion that depth analysis on quantitative 2D radiographs can capture 3D morphological variability and offer an alternative noninvasive method for the detection of atypical canal configurations.

The consistent changing of Xpar and Power Xpar values under zoomed panoramic conditions can be attributed to resampling processes intrinsic to digital magnification. Unlike optical zoom, digital magnification reconstructs intermediate pixel values through interpolation, redistributing gray-level information without adding anatomical data. Classical interpolation (bilinear or bicubic) tends to blur edges, whereas advanced edge-directed algorithms preserve directional continuity by estimating local covariance or gradient orientation [36, 37]. Recent DL-SR approaches try to rebuild high-frequency details through learned representations instead of solely interpolating them, which is different from typical digital zoom. But even these algorithmic developments might influence the grayscale pattern distributions and local textural characteristics, which should be kept in mind when looking at pixel-based quantitative descriptors [22]. While these methods enhance *perceived* resolution, they may also modify gray-level statistics directly relevant to texture-based morphometric parameters in dental imaging.

Numerically, such algorithms increase contrast along edges while amplifying variance in homogeneous regions [38, 39], thereby simulating fine structural heterogeneity that may be misinterpreted as genuine anatomical complexity. Clinically, this may improve visualization of canal boundaries or periapical regions yet al.so risks overestimating texture-derived metrics and hence canal complexity.

Within-subject comparisons between zoomed and non-zoomed measurements confirmed that digital magnification systematically inflated pixel-dependent quantitative parameters, particularly Pixel Count (*p* < 0.01 for 1023 × 496 non–C-shaped teeth; *p* = 0.002 for 1023 × 496 C-shaped teeth; *p* < 0.01 for 1023 × 593 C-shaped teeth), verifying that resampling augments matrix density without adding new anatomical content [36, 37, 39]. This effect was strongest in non–C-shaped teeth, where Xpar also differed significantly (Z = − 2.172, *p* = 0.029), suggesting that interpolation artifacts become more apparent in morphologically simple structures. Conversely, in anatomically complex roots, true heterogeneity masked pixel-added variance—consistent findings that edge-directed interpolation enhances local contrast at the expense of quantitative accuracy [36, 37, 39].

The present findings provide clinically relevant insight into the impact of resampling effects in panoramic radiography. Although digital zoom improves visual magnification, interpolation-based resampling modifies pixel intensity distribution and grayscale transitions, potentially influencing quantitative texture features and threshold-based assessments. In clinical practice, these thresholds should be interpreted within the context of the original-resolution image to minimize resampling-related bias. A practical approach would involve initial evaluation of native-resolution images, followed by correlation with clinical findings. In cases where quantitative values fall within borderline ranges or diagnostic uncertainty persists, clinicians should avoid relying solely on digitally magnified images and consider confirmatory imaging, such as periapical radiography or cone-beam computed tomography when clinically indicated. This workflow facilitates the integration of quantitative image analysis into routine diagnostic decision-making while maintaining patient safety and diagnostic accuracy.

Beyond the perceptual domain of the clinician, the implications of digital enhancement extend to artificial intelligence. Because digital zoom reconstructs pixel values by interpolating, it effectively creates synthetic visual data that is not consistent with the real anatomical structures. A learning algorithm trained on such resampled images may therefore misinterpret these artificial gradients as genuine morphological features, leading to biased feature extraction or false-positive classifications. In this context, the finding in this study that the Power Xpar metric remained highly discriminative even under the noise of zoom-induced interpolation may be of potential relevance for AI-enabled diagnostic systems. It may suggest that resolution-friendly quantitative descriptors, which are pixel-based metrics demonstrating relative stability across different native resolutions and under interpolation-induced grayscale variation, may help reduce susceptibility to resampling-related artifacts and contribute to more reliable feature representation in automated image analysis systems.

Regression and ROC analyses further suggested that image resolution, not magnification, appears to be more critical factor for diagnostic reliability. Although the zoom increased the metrics based on the pixels, it did not improve the overall model discrimination (AUC_zoomed = 0.723; AUC_non-zoomed = 0.745). This observation aligns with Øynes et al. [21], who differentiated *optical magnification*, which truly enhances spatial resolution by altering image geometry pre-acquisition, from *digital zoom*, which simply enlarges post-acquisition pixel matrices. Clinically, this implies that on-screen magnification tools may create an illusion of enhanced diagnostic detail, particularly in morphologically ambiguous areas.

Among all quantitative parameters, Power Xpar demonstrated the highest discriminative performance (AUC = 0.917 zoomed; 0.950 non-zoomed), suggesting its role as a resolution-tolerant descriptor that captures authentic high-frequency texture rather than interpolation artefacts. Importantly, a distinction should be made between bivariate discriminatory performance and multivariable modeling behavior. While ROC analysis reflects the isolated diagnostic capacity of individual texture parameters, multivariable logistic regression evaluates their independent predictive contribution under covariance conditions. In the present analysis, the regression models demonstrated only moderate discrimination and limited overall statistical significance, with pixel size emerging as the only consistently significant independent predictor. This finding likely reflects the influence of intrinsic spatial resolution on grayscale variance and texture computation rather than a direct biological association with canal morphology. As pixel size represents a technical imaging parameter, its statistical behavior should primarily be interpreted in relation to image formation characteristics rather than as evidence against the clinical relevance of texture-based metrics. The limited statistical significance observed in the multivariable models may also be partly attributable to the exploratory nature of this pilot study and the relatively limited effective sample size in relation to model complexity. Insufficient sample size relative to the number of candidate predictors is known to increase the risk of model overfitting, instability, and optimism in prediction modeling [40, 41]. Under such conditions, strong individual discriminatory signals may not necessarily translate into independent multivariable effects. Taken together, these findings suggest that although certain texture-based parameters demonstrate promising discriminatory performance, their independent predictive contribution requires validation in larger cohorts with more robust statistical power. Within the MediXpar analytical framework, further refinement and validation of Power Xpar–based panoramic texture analysis may enhance the consistency and objectivity of endodontic diagnostics in future clinical applications.

Nevertheless, several limitations should be acknowledged. The heterogeneity of imaging protocols may have introduced subtle gray-level bias between panoramic and CBCT datasets, and the absence of histologic validation prevents correlating radiographic heterogeneity with true tissue composition. The retrospective design constrained control over exposure and acquisition standardization. The sample size, particularly for patients with paired imaging modalities, was modest, and minor inter-system variations in detector performance could have influenced gray-level statistics. Furthermore, the dual-modality inclusion reduced the number of cases suitable for cross-validation. A further drawback is that the restricted quantity of diagnostically acceptable periapical radiographs in the retrospective archive precluded a statistically reliable comparison among the three imaging modalities (CBCT, panoramic, and periapical). This study employed a single-metric ROC approach rather than more contemporary radiomics or deep learning frameworks. However, its primary objective was not to develop a ‘black box’ Deep Learning model, but rather to explore which fundamental, interpretable pixel-based metrics (MediXpar) are associated with the anatomical complexity of C-shaped canals. Such basic image-processing analyses can serve as a complementary foundation for future Explainable AI models by revealing which measurable image features underpin the morphological variability observed in endodontic anatomy [42–44]. These limitations need careful interpretation. Even though the sample size met the minimum requirement set by the priori power analysis, the relatively small number of radiographs (*n* = 43) may still limit the generalizability of the findings. The low prevalence of C-shaped canals in the Turkish population (approximately 6%) inherently restricted the number of eligible cases available for this retrospective evaluation [6]. Also, the uneven distribution of C-shaped and non–C-shaped cases at different pixel resolutions (especially in the 1023 × 593 group) may have influenced the resolution-based ROC analyses and could have resulted in higher AUC estimates in smaller groups. While the stratified approach was intended to provide a detailed evaluation of resolution-dependent performance, this imbalance reinforces the exploratory nature of the present findings. Therefore, resolution-specific diagnostic indicators, particularly those related to Power Xpar, should be interpreted with caution.

Future prospective, multi-institutional studies with standardized imaging protocols are essential to validate the robustness and reproducibility of the present results. Such investigations should include multicenter validation studies involving larger and more demographically diverse populations to confirm the generalizability of these findings across different geographic and clinical settings. As an initial step, replication studies using the same imaging systems and acquisition parameters would help ensure methodological consistency and strengthen internal validation. Additionally, using data from different CBCT and panoramic machines that work under similar technical conditions would help determine if texture-based measurements, especially Power Xpar, stay reliable across different technologies and settings. Increasing both the sample size and device diversity would enhance external validity and provide a broader understanding of diagnostic performance. Furthermore, the incorporation of CBCT, panoramic, and periapical radiographs in subsequent research would facilitate direct cross-modality comparisons and enhance the clinical reliability of quantitative texture analysis.

From a clinical standpoint, the findings convey a clear principle: zooming may enhance visibility but not veracity. Diagnostic accuracy in panoramic radiography appears to depend chiefly on intrinsic resolution and robust quantitative descriptors rather than post-acquisition enlargement. Power Xpar, by demonstrating exceptional discriminative stability, may provide a pathway toward reproducible, data-driven diagnostics, ensuring that MediXpar can function as an analytical adjunct that transforms panoramic interpretation from a subjective visual process into an objective quantitative methodology. In practical endodontic settings, such quantitative thresholds may function as decision-support indicators during pre-treatment assessment. Elevated Power Xpar values (specifically ≥ 119.07 at native resolution) may alert clinicians to an increased likelihood of complex canal configurations when panoramic radiographs are routinely obtained. This information may assist in refining access cavity design, optimizing irrigation strategies, and evaluating the potential need for adjunctive CBCT imaging in anatomically ambiguous cases. Integration into the diagnostic workflow does not replace conventional clinical judgment but may complement it by providing objective and reproducible metrics that support treatment planning.

These findings also carry potential implications for forensic applications in which pulp-related radiographic parameters are used for age estimation. C-shaped canal systems may present distinct dentin–pulp complex volumetric configurations compared with conventional canal anatomies [33]. If such anatomical variations are not recognized, pulp-based age estimation models could theoretically be influenced by structural differences unrelated to chronological aging. In legal contexts where age thresholds bear significant implications, careful interpretation of quantitative radiographic biomarkers is essential to minimize potential sources of systematic bias. Although the present study was not designed to directly investigate age estimation, acknowledging morphological variability remains important when translating quantitative radiographic metrics into broader clinical or forensic practice.

## Conclusion

Among the MediXpar-derived quantitative parameters, Power Xpar demonstrated the highest discriminative capacity in ROC analysis and clinically meaningful results for identifying C-shaped canal morphology on panoramic radiographs. By applying depth analysis to commonly used 2D radiographs, MediXpar has the potential to become a reliable, resolution-independent alternative diagnostic platform. While digital magnification enhances visibility, it does not improve, and may distort quantitative diagnostic information. The diagnostic accuracy of texture analysis is fundamentally dependent on intrinsic image resolution. At higher resolutions, the Power Xpar metric proved to be a stronger discriminator for C-shaped canal morphology. This suggests that MediXpar, with adequate image acquisition quality, is a reliable tool for quantitative endodontic evaluation and offers a valuable complement to conventional radiographic interpretation.

## Data Availability

Due to patient privacy and ethical regulations, the datasets used in this study cannot be made publicly available. Data may be provided by the corresponding author upon reasonable request and with appropriate approvals.
